# ‘That is a Ministry of Health thing’: Article 5.3 implementation in Uganda and the challenge of whole-of-government accountability

**DOI:** 10.1136/tobaccocontrol-2021-057049

**Published:** 2022-01-25

**Authors:** Denis Male, Rob Ralston, Kellen Nyamurungi, Jeff Collin

**Affiliations:** 1 Department of Food Technology and Nutrition, School of Food Technology Nutrition and Bioengineering, Makerere University, Kampala, Uganda; 2 Global Health Policy Unit, Social Policy, School of Social and Political Science, University of Edinburgh, Edinburgh, UK; 3 School of Public Health, Makerere University, Kampala, Uganda

**Keywords:** low/middle income country, tobacco industry, global health, public policy

## Abstract

**Introduction:**

While Uganda has made legislative progress towards implementing Article 5.3 of the WHO Framework Convention on Tobacco Control (FCTC), ongoing challenges in minimising tobacco industry interference have not been adequately explored. This analysis focuses on understanding difficulties in managing industry engagement across government ministries and in developing effective whole-of-government accountability for tobacco control.

**Methods:**

Interviews with Uganda government officials within the health sector and beyond, including in Ministries of Trade, Agriculture and Revenue.

**Results:**

The findings indicate substantial variations in awareness of Article 5.3, its norm and practices across government sectors. The data suggest ambiguity and uncertainty about accountability for Article 5.3 implementation, with policy makers in departments beyond health often uncertain about obligations under the FCTC. Second, we highlight how responsibility for Article 5.3 implementation and the obligations incurred are widely seen as restricted to the Ministry of Health. Third, competing mandates and perceived difficulties in reconciling health goals with economic growth are shown to impact on accountability for tobacco control. Yet, importantly, the data also demonstrate enthusiasm in some unexpected parts of government for actively engaging with Article 5.3 and for promoting greater intersectoral coordination.

**Conclusion:**

This paper demonstrates the intrinsic challenges of developing whole-of-government approaches, highlighting considerable uncertainty and ambiguity among decision makers in Uganda about tobacco control governance. The analysis points to the potential for Uganda’s national coordinating mechanism to help reconcile competing expectations and demonstrate the importance of Article 5.3 beyond health actors.

## Introduction

The obligation in Article 5.3 of the WHO Framework Convention on Tobacco Control (FCTC) to protect public health policies from tobacco industry interference^1^ is widely recognised as ‘the backbone of the Convention’^2^ and improved adherence was endorsed by the WHO FCTC Impact Assessment Expert Group as the ‘single highest priority’ to advance tobacco control internationally.^3^ Yet levels of Article 5.3 implementation have been consistently low^4^ despite a sense that, given detailed guidelines and technical reports, countering industry interference ‘should be simple’.^5^ While tobacco industry actions are undoubtedly central to any explanation, difficulties in implementing Article 5.3 need to be understood within the context of broader governance challenges in developing coordinated and coherent approaches to tobacco control.^6 7^

The FCTC explicitly recognises the need for coordinated^8 9^ tobacco control governance across ministries. This whole-of-government approach is specified in Article 5.1, requiring Parties to develop comprehensive multisectoral national tobacco control strategies.^1^ In support of this, Article 5.2 requires Parties to establish a national coordinating mechanism (NCM) for tobacco control to promote coherent governance for health across ministries and government sectors. These commitments to coordination sit alongside Article 5.3 and its implementation guidelines^10^ that recognise the potential for corporate actors to undermine the FCTC^11^ based on a fundamental conflict between public health and tobacco industry interests.

The implementation guidelines^10^ outline a distinctive model of health governance^12^ including requirements to limit interactions with the tobacco industry, reject partnerships and avoid preferential treatment. Taken together, the general obligations under Article 5 aim to promote a coherent whole-of-government approach to tobacco control, seeking to reconcile competing interests and mandates within government and to protect policy making from tobacco industry within and beyond health departments. While the tobacco control literature has increasingly recognised the significance of institutional and bureaucratic dimensions to whole-of-government approaches, which necessitate government agencies working across portfolio boundaries to achieve a shared goal,^8 13–15^ these have not to date been explored via a primary focus on Article 5.3 and efforts to manage tobacco industry interference.

Accountability is a necessary condition of effective and legitimate governance, referring here to the mechanisms and practices designed to influence how institutions operate so that actors are made answerable for their conduct.^16 17^ In this paper, we assess to what extent Article 5.3 is understood within sectors and government departments in which policy goals and practices may be shaped by their respective mandates and accountability relations.^18^ Differing institutional mandates can imply competing or conflicting expectations or responsibilities across ministries and hence create or maintain policy tensions.^19 20^ Uganda’s 2015 Tobacco Control Act (TCA) is recognised as an example of good legislative practice in advancing Article 5.3 implementation.^21^ Delayed until May 2017 due to a legal challenge brought by the British American Tobacco Uganda (BATU),^5 6 22–24^ the TCA includes several key measures aimed at implementing Article 5.3. Its preamble includes commitments to ‘insulate tobacco control policies, laws and programs from interference by the tobacco industry’ and to ‘strengthen coordination, partnerships and collaborations for tobacco control’,^25^ and the Act draws on several Article 5.3 implementation guidelines^10^ via provisions to protect public health policy from the vested interests of the tobacco industry, ensure transparency of interactions between government and industry and restrict preferential treatment to the tobacco industry. However, the TCA also strikingly omits guideline recommendations to ‘raise awareness about the harmful nature of tobacco products and about tobacco industry interference with Parties’ tobacco control policies’ (table 1). In relation to Article 5.2, the TCA provides for an NCM tasked with ‘effective implementation’ of the FCTC and of ‘its implementing guidelines and protocols’,^25^ and specifies the participation of ministries including Trade; Environment; Agriculture; and Gender, Labour and Social Development.

**Table 1:** A comparison of guidelines to limit industry interference in public policy

WHO guidelines for implementation of FCTC Article 5.3	Uganda Tobacco Control Act 2015	Extent of fit
Raise awareness about the addictive and harmful nature of tobacco products and about tobacco industry interference with Parties’ tobacco control policies.		Omitted
Establish measures to limit interactions with the tobacco industry and ensure the transparency of those interactions that occur.	Ensure there is transparency in the interactions of government with the tobacco industry. The record and documents related to the interactions, communications and contacts held between the government and the tobacco industry shall be transparent and open to the public. A person, body or entity that contributes or may contribute to the formulation, implementation, administration, enforcement or monitoring of public health policies on tobacco control shall not interact with the tobacco industry except where it is strictly necessary for the effective regulation of the tobacco industry.	Broadly consistent
Reject partnerships and non-binding or non-enforceable agreements with the tobacco industry.	Prohibition on partnerships and endorsements of the tobacco industry. Any non-binding or non-enforceable agreement, memorandum of understanding, voluntary agreement or tobacco industry code of conduct in the place of legally enforceable tobacco control measures. Proposals, drafts or offers of assistance with the development or implementation of any tobacco control policies.	Broadly consistent
Avoid conflicts of interest for government officials and employees.	Prevention and management of conflict of interest: A person who contributes or may contribute to the formulation, implementation, administration, enforcement or monitoring of public health policies on tobacco control shall not engage in any occupational activity that may create a conflict of interest.	Broadly consistent
Require that information provided by the tobacco industry be transparent and accurate.	Reporting standards for tobacco industry to the Tobacco Control Committee.	Broadly consistent
Denormalise and, to the extent possible, regulate activities described as ‘socially responsible’ by the tobacco industry, including but not limited to activities described as ‘corporate social responsibility’.	Prohibition on partnerships and endorsements of the tobacco industry. Partnership of any kind with the tobacco industry, including initiatives or activities of the tobacco industry described, characterised, implied or likely to be perceived as socially responsible.	Broadly consistent
Do not give preferential treatment to the tobacco industry.	Prohibition of incentives or privileges to tobacco businesses.	Broadly consistent
Treat state-owned tobacco industry in the same way as any other tobacco industry.		Omitted

FCTC, Framework Convention on Tobacco Control.

In this paper, we examine the implementation of these legislative commitments, exploring awareness of and engagement with Article 5.3 across government sectors. We identify accountability for tobacco control as a key challenge for tobacco control, amidst broader ambiguity and uncertainty about the scope of obligations and of administrative and legal responsibilities among Ugandan government officials.

Drawing on in-depth interviews with policy officials across government sectors, this paper focuses on implementation of Article 5.3 to examine the accountability for tobacco control in Uganda and difficulties entailed in developing whole-of-government approaches. The findings highlight three distinctive challenges for tobacco control governance: (1) variable awareness, ambiguity and uncertainty across government sectors about Article 5.3; (2) differing expectations of departmental responsibilities for tobacco control policy; and (3) tensions with broader norms, notably in trade and agriculture, emphasising economic growth and often favouring the tobacco industry. While such varying expectations across different government sectors pose a significant challenge for tobacco control governance, we explore the potential for increased familiarity with Article 5.3 to enhance and broaden accountability.

## Methods

This paper draws on 35 semistructured interviews with representatives from across the Ugandan government, with policy officials from the Ministry of Health (MoH) (n=4), Ministry of Agriculture, Animal Industry and Fisheries (MAAIF) (n=2), the Ministries of Trade (n=3), Finance (n=2), Gender, Labour and Social Development (n=2), Education (n=2) and of Water and Environment (n=2), the National Environment Management Authority (n=2), National Planning Authority (n=1), Uganda Revenue Authority (n=1), National Bureau of Standards (n=2), Judicial Service Commission (n=1) and Kampala Capital City Authority (n=1), in addition to interviews with representatives of Non-Governmental Organisations (NGOs) (n=10).

KN and DM developed an initial list of interviewees based on in-depth knowledge of tobacco control policy in Uganda. Interviewee selection was guided by ‘snowball’ sampling^26^ using professional networks and recommendations made by other interviewees. Interviewees comprised senior and mid-ranking civil servants, law officers, policy makers and advocates, ensuring that diverse perspectives and experiences of tobacco control policy making were captured in the data. Interviews were semistructured, using a thematic topic guide covering three thematic areas: awareness of FCTC Article 5.3 and its guidelines; interaction between government and the tobacco industry; and implementation of FCTC Article 5.3. In addition to the topic guide, printed copies of FCTC Article 5.3 guideline recommendations were brought to interviews. In addition to serving as an aide-mémoire for interviewers, this document often performed a more interactive role in enabling interviewees with limited awareness of Article 5.3 to engage with its provisions.

Interviewee selection was coordinated by DM, KN and RR, with interviews conducted by four research assistants at Makerere University School of Public Health over the period July to August 2019. A 2-day workshop on Article 5.3 was conducted in Kampala by RR, KN and DM in June 2019, in which research assistants were sensitised to Article 5.3 and its guideline recommendations, and provided training on the topic guide. Interview length varied between 15 and 60 min, with most interviews being around 35 min. Interviewees reviewed and signed consent forms enabling digital recording and allowing data to be used in research publications. Interviews took place in private rooms with only the interviewee and interviewer(s) present, and were conducted in English, transcribed and subsequently anonymised. All transcripts were coded in NVivo 12 qualitative software using an analytical framework developed iteratively via multiple rounds of descriptive and conceptual coding. This entailed creating descriptive codes which were then contextualised using key governance concepts (notably accountability and coordination). In addition, the paper draws on key policy documents, including the 2015 Uganda TCA and National Tobacco Committee Guidelines.

All interview transcripts were coded by DM, and RR with input from KN and JC. Preliminary findings were reviewed at a Tobacco Control Capacity Programme consortium meeting in Addis Ababa, Ethiopia, in February 2020. The results and analysis were subsequently developed via coordination calls between DM, RR, KN and JC. The research obtained ethical approval from both Makerere University School of Public Health and the University of Edinburgh.

## Results

### Awareness, ambiguity and uncertainty

The interview data indicate the existence of considerable uncertainty among Ugandan government officials about accountability for Article 5.3 and tobacco control more generally. This was reflected in MoH officials questioning the extent of awareness and understanding of Article 5.3 beyond the health sector, and also a broader sense of ambiguity among interviewees about departmental responsibilities for implementing tobacco control measures.

The data suggest that policy officials working across non-communicable disease programmes within the MoH were generally familiar with Article 5.3. For example, one interviewee explained how the passage of the 2015 TCA created opportunities to increase awareness among policy officials of Article 5.3 and its norm of minimising interactions with the tobacco industry:

In my department it is high—in the Ministry of Health—I don’t know about other sectors but here it is high because during that period when we were debating the law in parliament, we sensitized them, so I think I would say it is high.

This was echoed by other health sector interviewees, with one official stating that they could ‘confirm that in the MoH, people are actually very aware’. Yet high levels of awareness within government were seen as being largely confined to health officials, with one interviewee reflecting that ‘the awareness is there, but there are not many of us’. These interviewees expressed doubt about the familiarity of policy officials in other ministries with Article 5.3 and whether this impacted on their interactions with the tobacco industry. As one health official noted, ‘with regard to other sectors I don’t know whether they have been sensitized enough. I don’t know [how] they relate with the industry.’

This scepticism among health officials was consistent with data provided by interviewees from other departments. Several such officials were familiar with the difficult passage of Uganda’s tobacco control law and BATU’s attempts to obstruct it. Some interviewees in non-health departments also demonstrated a degree of awareness of the FCTC and its legal obligations. For example, a policy official working on trade described how their ‘ministry is obliged to make sure they follow the FCTC and support it […] because if Uganda has ratified the convention, that means we are bound to it’.

Importantly, however, the data suggest that such familiarity in other ministries had not extended to implementation of rules or practices to limit tobacco industry interference in policy making. When asked whether measures to manage government–industry interactions had been adopted in their department, interviewees from the Ministries of Trade and the Environment indicated that, ‘in my sector, there are no measures’ and that they had ‘not seen any measures implemented’. Moreover, interviewees in Ministries of Agriculture, Trade and Revenue seemed to have a rather vague sense of Article 5.3 and its guidelines. For example:

I have heard about the Framework [FCTC] but I have not really read it or [thought] about it.

I do not know the organizational rules of Article 5.3 […] I am not sure of how well this article has been implemented.

I have not looked at the law or if it is necessary that I must look at the law [in terms of] what it provides and what I am supposed to do within that law [in] our role as public servants.

### ‘That is a Ministry of Health thing’

The data also indicate that interviewees in other government departments saw responsibility for tobacco control as being restricted to the MoH. While several interviewees indicated awareness of broad governmental obligations arising from the FCTC, these were seen as being of limited relevance for the work of non-health departments. This was apparent in responses to questions about what tools could be used to govern interactions with the tobacco industry, with Article 5.3 commitments seen as peripheral or extraneous. For example:

I can’t answer that for sure. That should be answered by the Ministry of Health [they] are in the best position to answer what measures are in place.

[…] my work is not public health. This law is about tobacco control—our colleagues at the Ministry of Health understand this law differently. This law is alien to our daily [working] lives.

The view that tobacco control was external to their responsibilities was common across interviewees beyond the MoH. Importantly, it was articulated by interviewees who were clearly broadly supportive of tobacco control. As one interviewee put it:

We are aware of the framework convention, but I think this convention is one of those which is [limited] to the health sector. [We] don’t use it—if it was ‘normal’ law that we use every day, I would [follow it] but the principal actor is the Ministry of Health.

The perception that tobacco control governance was separate from everyday work and practices highlights the difficulties of developing a coordinated and multisectoral approach to FCTC implementation. The implications of this narrow perception of accountability in restricting the scope of Article 5.3’s relevance and implementation were neatly captured by one civil society interviewee, who suggested:

If you say it is [the responsibility of] the Ministry of Health it will be like all the other activities, whereby those who are in other areas—be it Agriculture or other agencies of government—they will look at it like they look at issues such as immunization and say that is a Ministry of Health thing. It has to be a comprehensive thing.

### Competing mandates: tobacco control and economic growth

The data suggest that scope for multisectoral coordination was constrained by a broader norm, particularly evident in the Ministries of Trade and Agriculture, that the tobacco industry should be supported as part of pursuing national economic growth. This norm was invoked by interviewees in these sectors and justified as promoting legitimate economic interests. One policy official from the Ministry of Trade noted that it was their ‘responsibility to protect’ businesses, while another trade official argued:

As a ministry, of course, there is a conflict of interest, but you have to support the economy. For us that is trade […] if you encourage the investors to come in and to come and support the growing of tobacco, it’s well and good. The ministry is oblige[d] to make sure that they follow the FCTC and support it, but that is what I know.

Despite this interviewee acknowledging the tensions between tobacco industry and health interests and the obligation of their department to align policy practices with the FCTC, this indicates that tobacco control remains viewed in some ministries as a competing priority with (and a potential threat to) economic development. In the above example, the interviewee contrasts obligations under the FCTC with the reality of institutionalised economic norms favouring tobacco industry interests. The implications of this position for engagement with the industry were bluntly stated by an interviewee from agriculture, who noted that there were ‘those of us who cannot limit interaction with the industry […] there is no way I will say [that the] tobacco industry don’t come for a meeting in MAAIF’.

### Enthusiasm from unlikely quarters

Finally, and alongside competing mandates across government departments, the data indicate enthusiasm in some unexpected parts of government for greater intersectoral coordination and for building sensitisation to Article 5.3. While several interviewees in ministries beyond health were aware of Uganda’s tobacco control law and the FCTC, the data highlight uncertainty and ambiguity about responsibilities for and implications of Article 5.3 implementation. As one interviewee put it, ‘civil servants know very little about it considering it binds them to a code of conduct.’

In this respect, the process of conducting interviews often seemed to have a sensitising function, creating a space for interviewees to actively consider the implications of Article 5.3 guidelines for their work. As one trade policy official reflects, having engaged with Article 5.3 guidelines in the course of the interview:

I have read this provision within the recommendations of Article 5.3, where we are not required to give preferential treatment to the industry. That will definitely link to the work we do on trade. And also, there’s a recommendation 2 within the same provision that we will not limit an interaction to ensure transparency for the industry and whatever is happening and ensure that there’s that balance between public policy and trade […] The FCTC does relate to our work, you know, in as far as ensuring that trade does of course affect our public policy interests relating to health.

Similarly, officials in other departments saw Article 5.3 as potentially helpful in giving institutional legitimation to their own existing unease about interactions with the tobacco industry. One interviewee from the Ministry of Gender, Labour and Social Development described having ‘reservations about the [tobacco] industry, so the [tobacco control law] was a welcome proposal for me to see it coming into force […] if you have a copy, I will internalize it’.

The data suggest, however, that there have been limited opportunities for officials beyond health to become informed about Article 5.3 norms and rules. After Article 5.3 was described to them, one interviewee from the Department of Education responded: ‘That’s a very good article, but I am saying, who knows it?’ Another official highlighted the ‘need to induct people and sensitize them—to put it [Article 5.3] in documents and codes of conduct so that people know about it’. Others recommended prioritising efforts to sensitise relevant ministries to Article 5.3 and its guidelines. For example, an interviewee from the Ministry of Agriculture noted:

There could be a deliberate effort to make people understand it better […] you go out [and raise awareness of the law with] those actors who you think are instrumental to implementing it and sensitize them about it—they [will] understand what it is and become part of it.

The need for greater awareness raising efforts was linked by interviewees to the potential of governance mechanisms to facilitate greater intersectoral coordination. Interviewees described the recently created NCM as not having yet established itself as a key forum. For example:

The law was enacted and what, but I haven’t felt the visibility, and government doing its work, I have not felt it. For example, the tobacco control committee [NCM] was inaugurated sometime [in] April or May [2019] but we’ve had only one meeting because that’s where business is supposed to be generated, policies in relation to tobacco are supposed to come from there, that activity is not there.

Despite a formal requirement, codified in the 2015 TCA, for the NCM to meet ‘at least once every three months’,^25^ and the Act becoming operational in May 2017, this interviewee describes limited evidence of it promoting coordination across government sectors. This was attributed by interviewees to resource constraints. For example:

I think one of the key issues is to ensure that the [tobacco control] law is implemented is ensuring that the tobacco control committee is supported because it has the representation of different ministries and the prime minister—we need to ensure that it is supported […] why most government processes or good policies have failed is because the key bodies are not supported financially. If a committee is put in place and is not given the required support, then of course they will not perform their mandate effectively […] The Ministry [of Health’s] budget is very small, very little money and then you also find that other sectors have very little resources also. How are you going to implement the law?

## Discussion

This research explored Article 5.3 implementation in Uganda, focusing on examining accountability for health governance across government departments. It highlights key dimensions of accountability that appear to have shaped multisectoral coordination of tobacco control. First, that levels of awareness of Article 5.3 are high in the MoH but limited elsewhere, with understanding of being characterised by ambiguity and uncertainty beyond the health sector. Second, there is a widespread assumption that the responsibility for Article 5.3 implementation is restricted to the MoH, and that its requirements do not extend to other ministries. Third, Article 5.3 guidelines are viewed in some key ministries as conflicting with core responsibilities and economic norms. Yet the interview data also suggest encouraging enthusiasm in some unexpected parts of government, suggesting that greater familiarity with Article 5.3 and support in developing the NCM can consolidate progress made in addressing tobacco industry interference.

Uganda represents a significant context within which to explore these challenges, since its TCA is viewed as comparatively extensive in its coverage of Article 5.3 guidelines. The data presented here suggest that this recent legislative progress needs to be consolidated, particularly via efforts to increase awareness of the need to minimise government interactions with the tobacco industry beyond MoH and to support whole-of-government accountability. This need highlights the implications of the Act’s omission of provisions in the Article 5.3 guidelines for awareness raising and the resource implications of building effective and coordinated tobacco control governance. It is also consistent with reported breaches in observance of Article 5.3, notably including a national address by President Museveni that praised the Leaf Tobacco and Merchandise and the Meridian Tobacco for their contributions to fundraising efforts in the context of COVID-19.^27 28^ Such presidential endorsement of corporate social responsibility by tobacco companies powerfully illustrates the challenge of extending engagement with and accountability for Article 5.3 implementation across government. Accounts of limited progress in managing industry interference have understandably focused primarily on the corporate political activity of the tobacco industry^3 4^ but support the findings of Lencucha and colleagues^7 29 30^ in highlighting the importance of institutional factors in helping to explain coordination challenges within government. Building on their accounts of resource constraints as a barrier to intersectoral coordination, the findings suggest that divergent expectations of accountability have a significant impact on Article 5.3 implementation. Effective implementation of Uganda’s legislative commitments requires that tobacco control be established as a whole-of-government concern; to echo one interviewee, awareness and responsibility ‘has to be a comprehensive thing’ extending well beyond the narrow confines of the MoH.

The interview data highlight the ongoing significance of perceived tensions between tobacco control and economic growth. These remain remarkably durable for two decades since the World Bank’s landmark report *Curbing the Epidemic*,^31^ and despite Uganda-specific evidence demonstrating that the local costs of tobacco-related illnesses in Uganda dramatically outweigh benefits from industry-generated employment and tax revenue.^32^ Policy conflicts between health-focused and economy-focused government actors in African contexts have been attributed to the interplay of entrenched economic development norms, the tobacco industry’s ability to shape policy discourses and structural divisions between sectors reflected in ‘bureaucratic silos’.^7^ A distinctive contribution of the current study is that it highlights routes towards addressing such conflicts by using Article 5.3 implementation as a basis for clarifying accountability expectations and promoting intersectoral coordination.

Data illustrating varying awareness of Article 5.3 obligations and competing perceptions of government–industry interactions across ministries may be unsurprising. We would attach particular significance, however, to data indicating enthusiasm for such provisions among government officials previously unfamiliar with provisions such as minimising interactions and promoting transparency. From the perspective of promoting coherent approaches to tobacco control, there is a clear value in officials from the Ministry of Trade recognising the relevance of such principles to their work, and of officials beyond health recognising Article 5.3 as providing a legitimating tool consistent with their existing disquiet about government relationships with the tobacco industry. Demonstrating such value and relevance requires an active commitment to awareness raising, but the (admittedly limited) experience of conducting this research suggests that such efforts may be effective. The very process of conducting interviews often seemed to have served a *de facto* sensitising function, creating a space for officials to actively consider the implications of Article 5.3 guidelines for their work.

Given the interdependence of general obligations under Article 5, promoting whole-of-government engagement with, and accountability for, Article 5.3 can serve as the basis for policy coordination and a powerful instrument to advance the achievement of wider tobacco control measures.^33^ Increasing recognition among Parties that strengthening governance obligations should be prioritised to advance FCTC implementation provides a promising basis for progress.^6^ The interview data offer a reminder, however, that activities such as awareness raising do have resource implications, and the significance of these measures requires that they be supported by governments, donor agencies and philanthropies. The tobacco industry’s high-profile use of corporate philanthropy to exploit Uganda’s financial challenges amid the COVID-19 pandemic in Uganda provides a cautionary note of how tobacco control governance can be undermined by resource constraints.

**What this paper adds**

- Uganda’s Tobacco Control Act 2015 includes comprehensive coverage of international guidelines for Article 5.3 of the WHO Framework Convention on Tobacco Control, but significant implementation challenges persist.
- A focus on accountability highlights institutional barriers to developing effective whole-of-government approaches to tobacco control governance.
- Our qualitative analysis of experiences of Article 5.3 implementation across diverse government ministries highlights variable levels of awareness; uncertainty and ambiguity about the scope of its applicability; a widespread assumption that responsibility is restricted to the Ministry of Health; and perceived conflict with economic norms in key ministries.
- The data indicate potential support for Article 5.3 principles in unexpected parts of government. Awareness-raising activities could clarify accountability expectations and help promote coherent governance across ministries via the recently established national coordination mechanism.

## Data Availability

No data are available. Not applicable.
